# MindGrab: A spectrally-motivated architecture for accessible deep learning in neuroimaging

**DOI:** 10.1016/j.neuroimage.2026.122074

**Published:** 2026-06-23

**Authors:** Armina Fani, Mike Doan, Isabelle Le, Alex Fedorov, Malte Hoffmann, Chris Rorden, Sergey Plis

**Affiliations:** a Tri-Institutional Center for Translational Research in Neuroimaging and Data Science (TReNDS), Georgia State University, Georgia Institute of Technology, Emory University, 55 Park Pl NE, Atlanta, GA, 30303, USA; b Emory University, 201 Dowman Dr, Atlanta, GA, 30322, USA; c Harvard University, Massachusetts Hall, Cambridge, MA, 02138, USA; d University of South Carolina, 1501 Pendleton St, Columbia, SC, 29208, USA

**Keywords:** Skull stripping, Neuroimaging, Dilated convolutions, Deep learning, Zero footprint AI, Omnimodal

## Abstract

Deployment complexity and specialized hardware requirements hinder the adoption of deep learning models in neuroimaging. We present MindGrab, a lightweight, fully convolutional model for volumetric skull stripping across the evaluated imaging modalities. MindGrab’s architecture is designed from first principles using a spectral interpretation of dilated convolutions, and demonstrates state-of-the-art performance on the tested benchmarks (mean Dice score across datasets and modalities: 95.9 ± 1.6), with up to 40-fold speedups and substantially lower memory demands compared to established methods. Its minimal footprint allows for fast, full-volume processing in resource-constrained environments, including direct in-browser execution. MindGrab is delivered via the BrainChop platform as both a simple command-line tool (pip install brainchop) and a zero-installation web application (brainchop.org). By removing traditional deployment barriers without sacrificing accuracy, MindGrab makes state-of-the-art neuroimaging analysis broadly accessible.

## 1. Introduction

The transformative potential of deep learning in neuroimaging is increasingly hampered by a critical, yet often overlooked, barrier: deployment complexity. While novel architectures demonstrate state-of-the-art performance, their practical adoption by clinicians and researchers is severely limited by immense technical requirements (Renton et al., 2024; Gronenschild et al., 2012). These models often demand specialized hardware like high-end NVIDIA GPUs, convoluted software installations, and command-line expertise, effectively excluding the vast majority of their intended users. This disparity creates a paradox where our most powerful analytical tools remain inaccessible for routine point-of-care or research applications.

The specialized hardware required for deployment of these models is often only available through cloud-based platforms, which introduces a separate, frequently insurmountable, obstacle: data privacy. Strict institutional policies and regulations, such as HIPAA, prohibit the transfer of protected health information (PHI) to third-party servers, rendering most cloud solutions unsuitable for clinical data (Plis et al., 2016; Kaissis et al., 2020). This leaves the field at an impasse: local deployment is too complex, and cloud deployment is too insecure for many institutions under current regulatory constraints, hindering progress and compromising the quality of downstream analyses.

This challenge is particularly acute for foundational preprocessing tasks like skull stripping– the removal of non-brain tissue from neuroimaging scans. For decades, the field has relied on classical methods like BET (Smith, 2002) and ROBEX (Iglesias et al., 2011), which, despite their utility, often struggle with variations in image contrast and quality. Deep learning models, typically based on large U-Net architectures (Ronneberger et al., 2015), offered a leap in accuracy. More recently, the challenge of generalizing across different scanners and modalities was elegantly solved by training models entirely on synthetic data (Billot et al., 2023; Gopinath et al., 2024). The state-of-the-art method, SynthStrip (Hoopes et al., 2022), leverages this strategy to achieve unprecedented robustness. However, its large, parameter-heavy architecture imposes many practical challenges. Its deployment requires navigating a fragile ecosystem of software dependencies– a task demanding a level of systems administration expertise that is orthogonal to clinical and scientific practice. Furthermore, its computational and memory footprint, while manageable on a dedicated workstation, renders the architecture unsuitable for deployment in resource-constrained environments such as web browsers and handheld devices, which are becoming ubiquitous in clinical and research settings. These hurdles create significant friction for adoption, effectively limiting the immediate use of such powerful tools by clinicians at the point of care and researchers focused on analysis, not software installation.

To resolve this tension between accuracy and accessibility, we present MindGrab, an extremely efficient deep learning model for skull stripping. MindGrab achieves Dice scores that are highly comparable to SynthStrip across evaluated datasets by favoring higher precision (fewer false positives) in its segmentation strategy. This accuracy is achieved with a model that has 95% fewer parameters, an efficiency that translates into dramatic real-world performance gains, including up to 40-fold speedups and substantially lower memory usage on both high-end GPUs and consumer-grade hardware, enabling novel deployment modalities such as in-browser execution via our BrainChop platform (Plis et al., 2024). This architecture was designed from first principles guided by a spectral analysis of dilated convolutions. We demonstrate that this design, which systematically reduces spatial frequencies, is key to MindGrab’s success by validating its performance against alternative dilation patterns. MindGrab thus provides the neuroimaging community with a tool that delivers competitive performance and is immediately usable without the traditional barriers of software installation or hardware dependency.

## 2. Methods

### 2.1. Model architecture and dilation schedule design

The design of dilated convolution architectures, such as MeshNet by Fedorov et al. (2017), has proven highly effective for patch-based analysis, where a receptive field of 69^3^ voxels is well-suited for processing sub-volumes. This strategy is suboptimal for single-pass, whole-volume analysis, as it risks stitching artifacts and cannot leverage global context. Simply extending traditional dilation schedules (Yu and Koltun, 2016) to cover a full volume (e.g., 256^3^) is inefficient and offers no clear guidance for creating a memory-light model. This motivated our move from a purely spatial receptive field analysis to a spectral perspective.

**Fig. 1.**
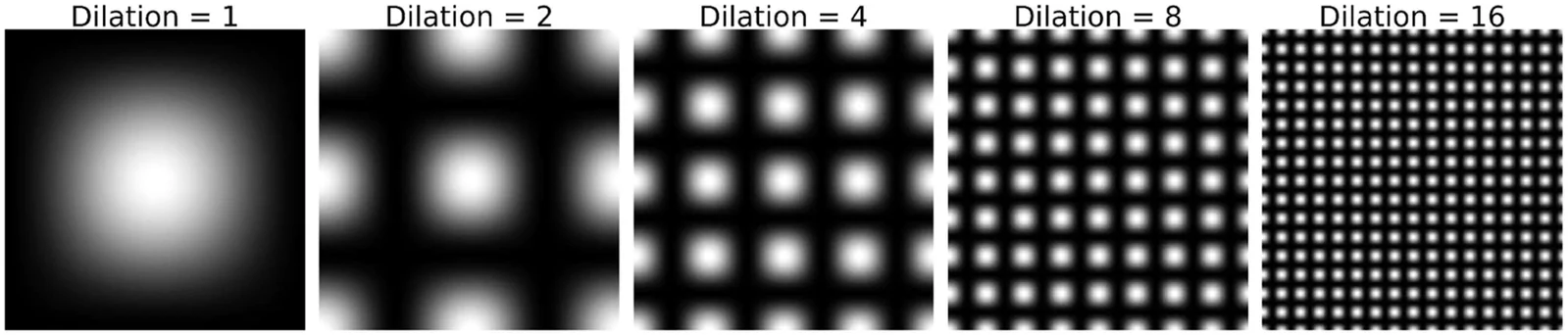
k-Space magnitude envelopes of a 3 × 3 kernel with different dilations. Increasing dilation produces progressively denser spectral replication, broadening the range of spatial frequencies the filter can interact with while keeping the kernel size unchanged.

This spectral view provides a principled design path. In the frequency domain, dilation acts as a tuning mechanism. Introducing gaps between kernel weights creates a periodic filter that replicates its Fourier response across k-space (see Fig. 1). This allows a single, small kernel to become sensitive to multiple, widely separated frequency bands without increasing its parameter count. Increasing the dilation rate creates a denser tiling of these sensitivity bands, broadening the range of spatial frequencies the layer can interact with. In essence, the dilation schedule determines which frequency bands the network can “see,” while the learned weights determine the response within those bands. Although the dilation schedule produces a multiscale receptive-field hierarchy, it is not meant to represent a diffusion process (Perona and Malik, 1990) or a classical SIFT-style scale-space pyramid (Lowe, 2004); instead, it is introduced to control contextual integration and frequency coverage within a learned convolutional architecture. This principle is the foundation of our architectural design.

To exploit this property, we group layers (each a sequence of convolution, normalization, and Gaussian Error Linear Unit (GeLU) activation function (Hendrycks and Gimpel, 2023) into 5-layer dilation blocks that act as controlled spectral bottlenecks. Two complementary schedules are considered:

- Increasing: 1→2→4→8→16 (denoted ◂) — progressively broadens frequency support (sharpening).
- Decreasing: 16→8→4→2→1 (denoted ▸) — progressively contracts frequency support (blurring).

Because all layers are isometric (have the same spatial dimensions) and use the same channel count, the only variable that changes is the dilation pattern itself. This allows us to attribute performance differences directly to spectral structure rather than depth, capacity, or skip connections. This ordering of dilations produces a multiscale flow similar in spirit to a Laplacian pyramid, achieved without changing image resolution.

MindGrab is built from five consecutive decreasing blocks (▸▸▸▸▸), each containing five 3 × 3 × 3 convolutions with 15 channels, followed by a final 1 × 1 × 1 projection layer (26 layers total). The network uses parameter-free per-volume z-scoring and no biases. During inference, only one activation map is stored at a time, enabling deployment on memory-limited hardware and direct in-browser execution as showcased in our BrainChop platform.

### 2.2. Synthetic data generation for training

MindGrab was trained *exclusively on synthetic data* generated with Wirehead (Doan and Plis, 2025), a data generation pipeline leveraging SynthSeg (Billot et al., 2023) to continuously produce diverse synthetic (brain image, label) pairs. SynthSeg begins with an anatomical label map and applies a series of randomized augmentations, including spatial deformations, intensity variations, resolution randomization, and simulation of imaging artifacts such as blurring and noise. This process enables the model to learn domain-agnostic features, making it highly robust to variations in real-world scans. Our input label set to Wirehead included 171 volumes, each with 39 standard anatomical FreeSurfer and non-brain labels. This included 131 label maps from the publicly available SynthStrip dataset (Hoopes et al., 2022) plus 40 cropped variants generated by truncating superior and inferior brain areas to simulate scans with sharp cutoffs.

All synthetic data were preprocessed with 2nd-98th percentile quantile normalization. Brain-specific labels were merged into a binary mask with smoothed edge boundaries. MindGrab was trained on approximately 250k synthetic samples using the Adam optimizer, soft-dice loss, 50 cycles of OneCycle-LR, and a batch size of one.

### 2.3. Evaluation datasets

The validation dataset was derived from the multimodal benchmark compiled for SynthStrip evaluation in the original SynthStrip publication (Hoopes et al., 2022), comprising 606 adult images from eight public datasets (Biomedical Image Analysis Group; Greve et al., 2021). This cohort spans healthy and pathological populations, multiple acquisition protocols and field strengths, and diverse imaging contrasts, providing a heterogeneous dataset for robust skull stripping evaluation. The original SynthStrip benchmark also includes a subset of infant data, which we exclude from this evaluation. MindGrab was trained exclusively on synthetic data generated from adult anatomical distributions, and no infant labels were included in the Wirehead input set. Infant brain imaging poses distinct challenges, including rapid developmental changes in morphology and reduced tissue contrast. Notably, even in the original SynthStrip study (Hoopes et al., 2022), performance on infant data was comparatively weaker, which subsequently motivated dedicated work on infant-specific skull stripping (Kelley et al., 2024). Given these domain-specific challenges, we restrict our analysis to adult datasets.

The IXI dataset contributed 50 T1-weighted (T1w), 50 T2-weighted (T2w), 50 proton density-weighted (PDw), 50 magnetic resonance angiography (MRA), and 32 diffusion-weighted imaging (DWI) scans. The FSM subset included 38 T1w, 36 T2w, 32 PDw, and 32 quantitative T1 maps (qT1). These subsets primarily consist of healthy adult brains across varied contrasts, enabling evaluation under intensity variability and reduced tissue contrast (e.g., PDw, qT1).

SynthStrip provided 43 pseudo-continuous ASL (PCASL) T1w scans and 43 2D echo planar imaging (EPI) acquisitions (Harms et al., 2018; Juttukonda et al., 2021). These data are low-resolution clinical stacks with a limited field-of-view that often crops the ventral region, thereby testing robustness to incomplete coverage and geometric artifacts.

Clinical stacks of thick image slices from the QIN glioblastoma dataset contributed 54 T1w, 39 T2w, and 17 T2-FLAIR volumes (Mamonov and Kalpathy-Cramer, 2016; Prah et al., 2015; Clark et al., 2013). This dataset comprises patients with newly diagnosed glioblastoma, scanned after surgery but prior to treatment, and therefore introduces pathological variation. The CERMEP-IDB-MRXFDG (CIM) database provided 20 brain CT and 20 PET scans of normal adult human subjects (Merida et al., 2021). These modalities extend evaluation beyond MRI: CT presents high bone intensity and limited soft tissue contrast, while PET exhibits low spatial resolution and high noise, both of which complicate accurate delineation of brain boundaries.

All samples were accompanied by a silver-standard reference brain mask generated for the original SynthStrip paper (Hoopes et al., 2022) using an ensembling. First, brain masks were acquired using each classical method evaluated in their study (ROBEX 1.1, BET from FSL 6.0.4, 3dSkullStrip from AFNI 21.0.21, BEaST 1.15, and the FreeSurfer 7.2 watershed algorithm (FSW)). A consensus mask was computed for each subject by taking the voxel-wise majority across the set of automatically generated masks. Consensus masks were manually refined. To extend the labels across modalities, the masks were propagated by rigidly aligning each subject’s T1w scan to the remaining image types using a robust registration procedure, with further refinement by hand.

We use the Human Connectome Project (HCP) Young Adult 1200-subject dataset as an auxiliary evaluation set, comprising 1,113 T1-weighted scans of healthy young adults with accompanying labels (Van Essen et al., 2013; Glasser et al., 2013). One scan was excluded due to quality control failure, resulting in a final sample size of 1,112. The ground-truth annotations retain the original 42 FreeSurfer-derived brain segmentations and include 13 additional non-brain labels (e.g. eyes, bone) generated using SimNIBS CHARM (Puonti et al., 2020). The final label files were constructed using an adult-adapted subset of the Medley curation approach, specifically its label harmonization and data construction logic rather than the full Medley pipeline (Le et al., 2026). We further include 1,068 T2-weighted scans rigidly registered to their corresponding T1w images using Broccolini (Rorden, 2026; Eklund, 2014), allowing the same label maps to be applied across modalities. All data were resampled to 256^3^ shape with 1-mm isotropic resolution.

## 3. Results

The following subsections evaluate the performance of MindGrab against baseline models across multiple imaging modalities in terms of accuracy, robustness, and computational efficiency. We also evaluate MindGrab⚡, which is the same model as MindGrab, but applied to cropped versions of the input, where the empty space around the head is discarded before application. We further isolate the contribution of dilation order and normalization strategy to quantify their roles in the model’s performance.

### 3.1. Comparison with existing models

We evaluate the similarity between computed and ground-truth brain masks for MindGrab, SynthStrip, ROBEX, BET, and MindGrab⚡ by reporting average Dice, precision, and recall scores. Where given, the significance of MindGrab scores is determined by two-sided Wilcoxon signed-rank significance tests.

The Dice comparison in Table 1 demonstrates that MindGrab achieves average Dice scores exceeding ROBEX and BET and highly comparable to SynthStrip. MindGrab significantly outperforms ROBEX across all datasets and is significantly better than BET in all but two cases: IXI DWI (no significant difference) and ASL EPI (BET performs better). Compared to SynthStrip, MindGrab is significantly better in four categories, shows no significant difference in three, and is significantly worse in nine, remaining within 3% of SynthStrip’s Dice score, indicating competitive overall performance.

**Fig. 2.**
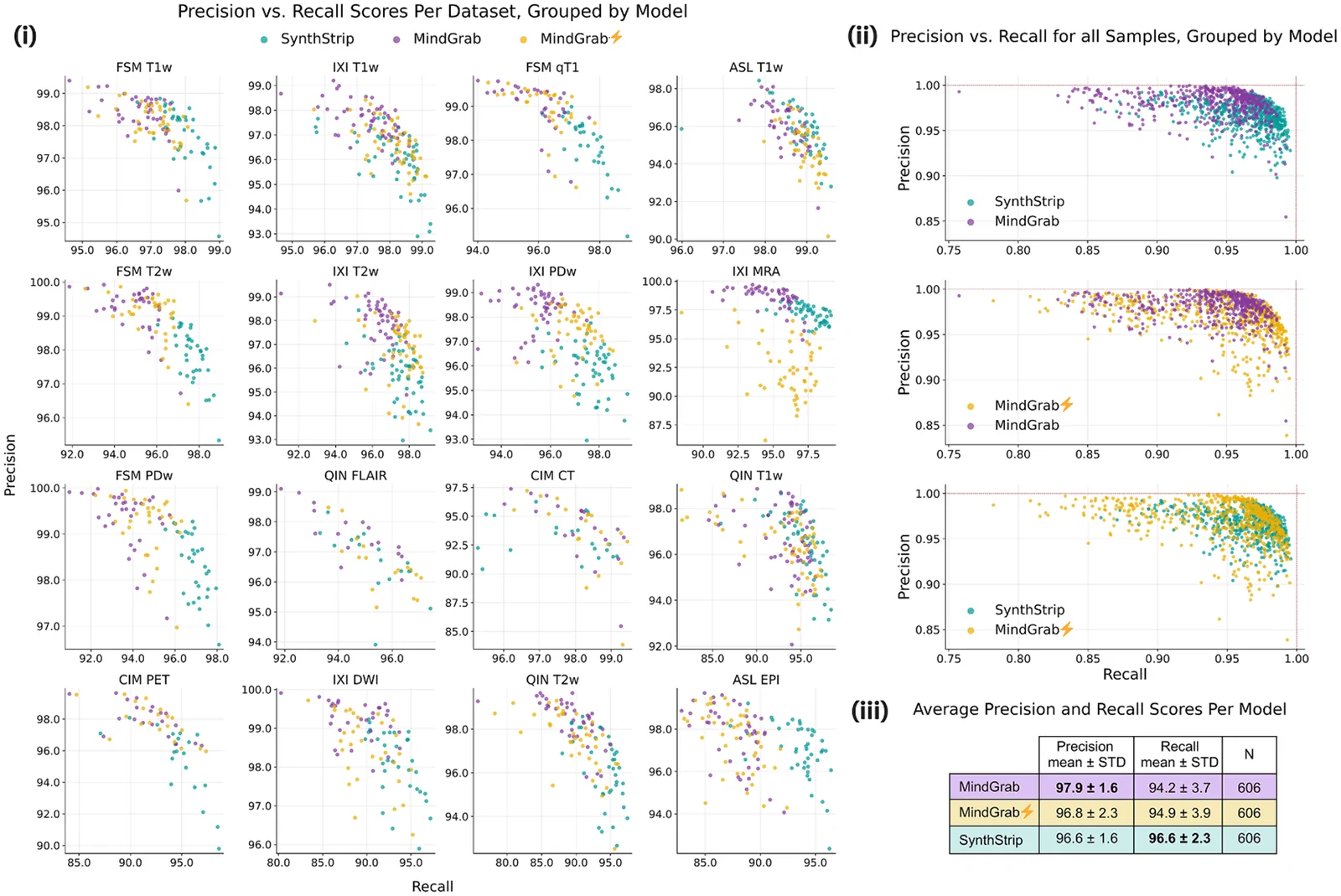
Precision and recall comparison across evaluation datasets. Panel (i) presents a scatter plot grid of precision (y-axis) vs. recall (x-axis) scores for the 16 evaluated datasets. Each dot represents a single sample, with SynthStrip scores in blue, MindGrab in purple, and MindGrab⚡ in yellow. MindGrab consistently displays higher precision, while SynthStrip generally achieves higher recall. MindGrab⚡ occupies an intermediate position, offering a balance between the two. Panel (ii) provides a global view of the precision-recall relationship by grouping all samples by model. The top graph in this sub-figure compares SynthStrip and MindGrab, the middle compares MindGrab and MindGrab⚡, and the bottom compares SynthStrip with MindGrab⚡. Panel (iii) is a summary table that presents the mean precision and recall (± standard deviation) and the total sample count for the three models, confirming the trends observed in the scatter plots. The highest score for each metric is bolded.

Fig. 2 gives insight into precision and recall trends for MindGrab, MindGrab⚡, and SynthStrip. MindGrab (purple) generally achieves higher precision scores, whereas SynthStrip (blue) demonstrates higher recall across the majority of datasets. Notable exceptions to this trend are observed in datasets CIM CT, ASL T1w, QIN T1w, and QIN FLAIR, where the performance relationship between the models is either more equivalent or less distinct. MindGrab⚡’s performance (yellow) typically falls between these two models, indicating a balance between the precision and recall trade-off.

This relationship is reinforced by the aggregate data presented in Panel (ii), which plots all samples by model. MindGrab trends towards higher precision, whereas SynthStrip favors higher recall (top subplot). MindGrab⚡ mostly follows MindGrab’s performance, with slight variability in precision and marginally higher recall. This positions MindGrab⚡ at a slightly higher precision and lower recall than SynthStrip. The quantitative summary in Panel (iii) further supports these observations. MindGrab has the highest precision of the three, with a score of 97.9 ± 1.6 compared to SynthStrip’s 96.6 ± 1.6 and MindGrab⚡’s 96.8 ± 2.3. SynthStrip has the highest average recall with a score of 96.6 ± 2.3, compared to MindGrab’s 94.3 ± 3.7, and MindGrab⚡’s 94.9 ± 3.9. (See supplementary Tables S1 and S2 for Mean Surface Distance and 95th Percentile Hausdorff Distance scores).

### 3.2. Qualitative evaluation of brain-mask boundaries

**Fig. 3.**
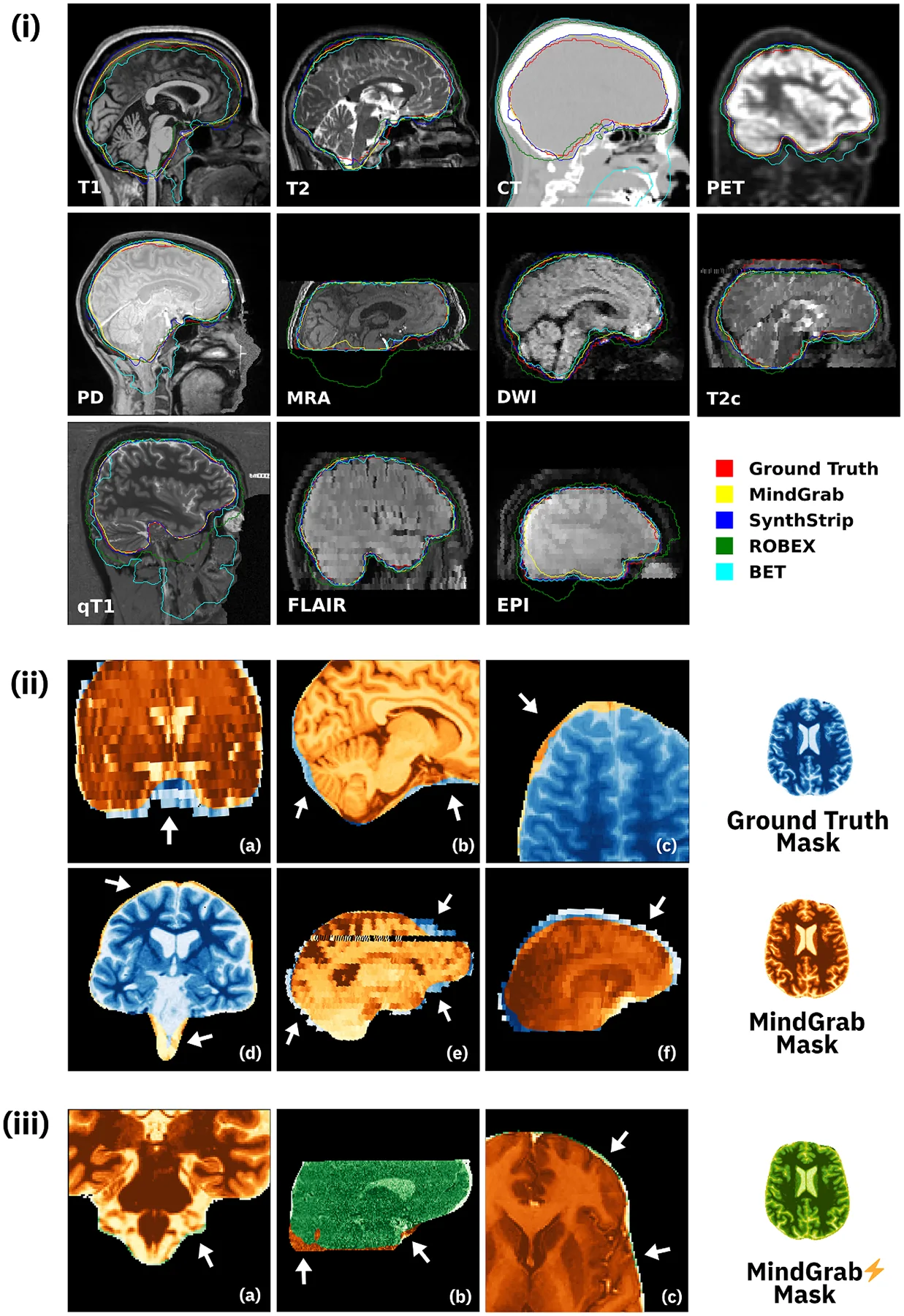
Qualitative comparison of skull stripping results across different methods and imaging modalities. In Panel (i), segmentation boundaries are displayed as colored contours overlaid on representative sagittal slices from different imaging modalities: MindGrab (yellow), SynthStrip (blue), ROBEX (green), and BET (cyan). Ground truth contours are shown in red. Note the variable performance of classical methods (ROBEX, BET) and the generally comparable accuracy between MindGrab and SynthStrip. Panel (ii) details instances of the rare MindGrab error by highlighting differences in ground truth and MindGrab mask overlap. MindGrab masks are orange, and ground truth masks are blue. MindGrab shows cases of both over- and under-segmentation. Panel (iii) compares MindGrab and MindGrab⚡, with MindGrab⚡ masks shown in green. While MindGrab⚡ generally shows minimal differences from MindGrab, it tends to under-segment in regions with sharp intensity transitions.

Panel (i) of Fig. 3 qualitatively compares MindGrab, SynthStrip, ROBEX, and BET through superimposed segmentation and the silver-standard ground-truth contours overlaid on input images. Classical methods like ROBEX (green) and BET (cyan) show variable performance, with notable instances of both over- and under-segmentation. SynthStrip (blue) demonstrates reliable boundary alignment but exhibits minor over-segmentation inferior to the medial prefrontal cortex. MindGrab (yellow) achieves comparable efficacy with reduced precision near high-contrast boundaries, as seen in the MRA example. Both SynthStrip and MindGrab fail to segment regions beyond abrupt intensity transitions, as seen superior to a dark artifact in the T2c image.

Panel (ii) details localized segmentation errors associated with MindGrab. Subpanels (a) and (f) show a conservative segmentation approach, characterized by a slight under-segmentation of the brain when it is in close proximity to the skull or dura. This behavior is also present in subpanel (b), where the arrows highlight a subtle under-segmentation of the cerebellum’s inferior boundary. Conversely, subpanels (c) and (d) reveal cases of slight over-segmentation, where the predicted mask is marginally larger than the ground truth.

Panel (iii) compares the segmentation outputs of MindGrab and MindGrab⚡. As shown in subpanels (a) and (c), the typical difference between the two models is minimal and often visually imperceptible. However, on more challenging datasets, MindGrab⚡ can exhibit instances of greater under-segmentation, as seen in subpanel (b). These larger deviations from the ground truth are particularly notable in images with artifacts that have sharp boundary transitions. This behavior is consistent with the lower precision observed in the quantitative analysis section earlier.

### 3.3. Computational efficiency analysis

**Fig. 4.**
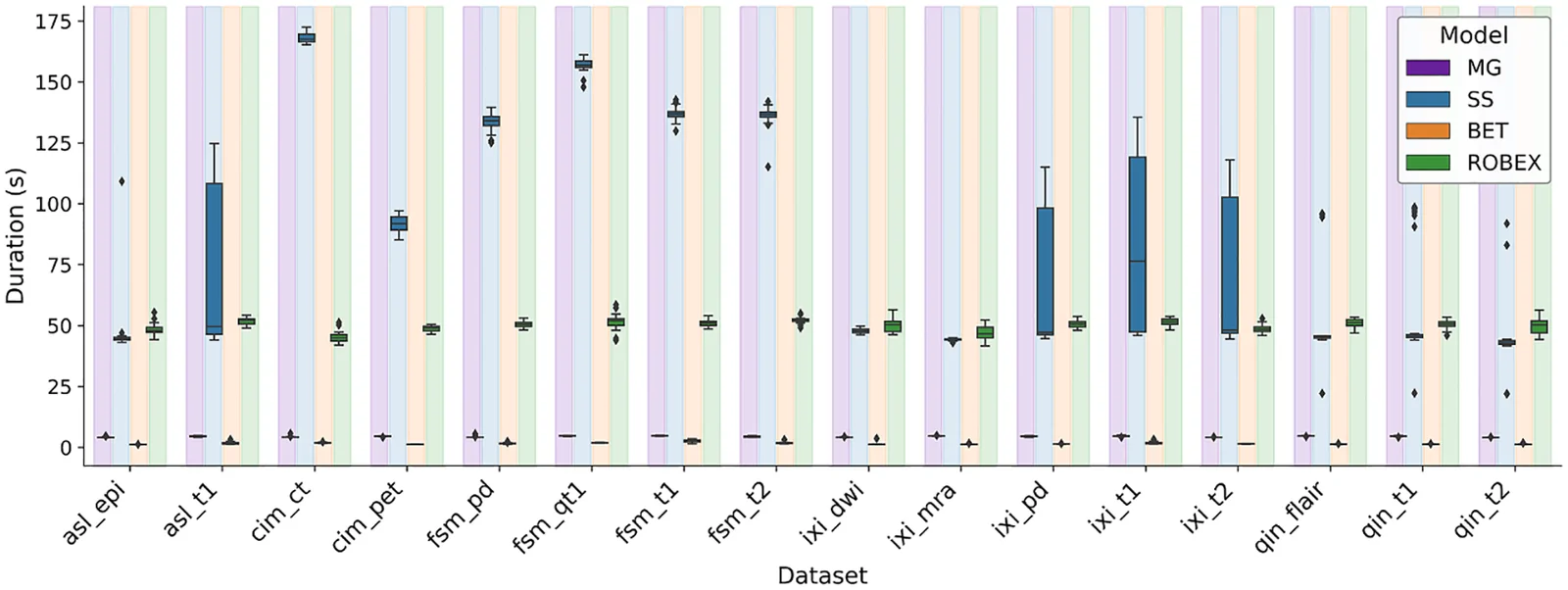
Runtime across datasets. Boxplots show runtime (seconds) for MindGrab (MG), SynthStrip (SS), BET, and ROBEX on each dataset. Muted vertical bands mark tool identity where distributions overlap. BET is the fastest tool, with MindGrab close behind. SynthStrip shows the most runtime spread. ROBEX and SynthStrip trade places as the slowest tool across datasets.

We report runtime for all four methods across the 16 SynthStrip datasets, reflecting typical end-to-end tool usage rather than controlled, hardware-normalized benchmarking. MindGrab, SynthStrip, and BET were executed on an Apple M2 system using their default configuration. ROBEX does not natively support Apple Silicon environments and was therefore executed on a Linux system with its default configuration. Fig. 4 displays runtime distributions for the evaluated datasets, with dataset on the x-axis and runtime (seconds) on the y-axis. MindGrab (MG) is shown in purple, SynthStrip (SS) in blue, BET in orange, and ROBEX in green. To improve readability, vertical shaded bands corresponding to each model are included behind the boxplots, as some distributions are too compact to distinguish by color alone. Runtime was measured per image using wall-clock time over the full pipeline, from NIfTI loading to output saving.

Across datasets, SynthStrip exhibits the greatest variability in runtime, with substantially longer execution time in several cases (e.g., ASL T1 and CIM CT). It is frequently the slowest tool, matched or exceeded in some datasets by ROBEX. In contrast, MindGrab and BET demonstrate consistently low runtime duration with tight distributions across all datasets. BET achieves the lowest runtimes overall with MindGrab closely following.

**Fig. 5.**
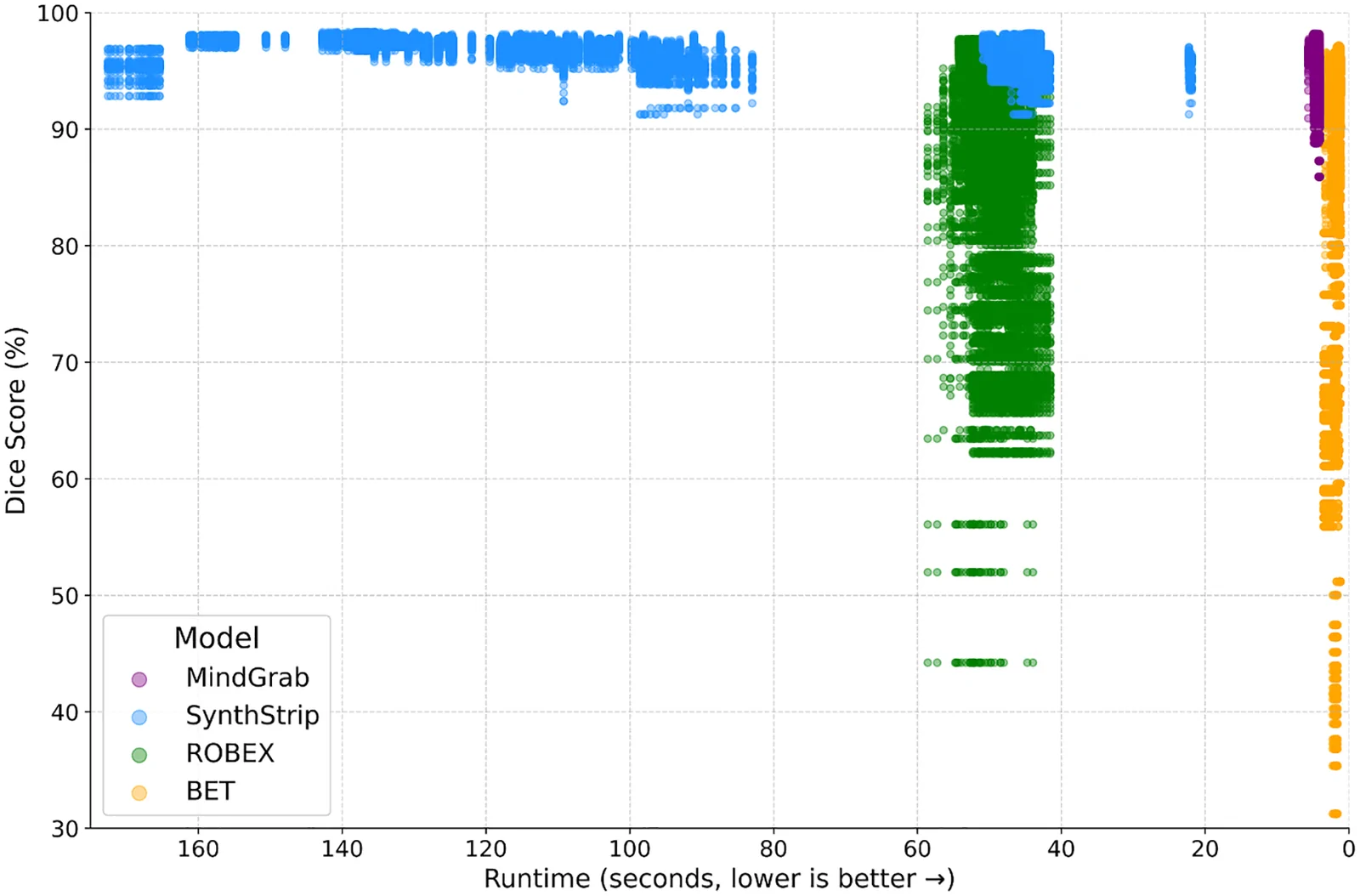
Dice-runtime trade-off across evaluated models. The x-axis shows runtime (seconds, inverted so faster methods appear to the right), and the y-axis shows Dice score. MindGrab (purple) forms a cluster at the top-right of the scatterplot characterized by low runtime and high Dice. Synthstrip (blue) achieves consistently high Dice scores but exhibits substantial variability in runtime. BET (orange) shows consistently low runtime but large variability in Dice performance. ROBEX (green) operates within an ~40–60 s runtime range but displays wide variation in Dice scores.

To contextualize these runtime results in relation to segmentation performance, Fig. 5 presents a joint view of runtime cost and accuracy. The scatterplot displays runtime on the x-axis (inverted, such that faster methods appear to the right) and Dice score on the y-axis for all samples in the SynthStrip dataset. Models are shown using the same color scheme as above. The most desirable point on the scatterplot is the upper right corner of zero runtime at 100% Dice score.

MindGrab occupies a compact region in the top-right of the plot, reflecting consistently high Dice scores (>85 and concentrated above 95) paired with low runtime (<10 s) and minimal variability. SynthStrip achieves similarly high and stable Dice scores but exhibits substantial variability in runtime, ranging from approximately 20 s to over 160 s. In contrast, BET demonstrates consistently low runtime but with wide variability in Dice score, spanning from poor performance (~30) to high-quality segmentation (>90). ROBEX shows stable runtime within the 40–60 s range, but similarly exhibits large variability in Dice performance, ranging from mid-40s to high 90s.

This visualization highlights the trade-offs between accuracy and runtime efficiency across tools. MindGrab uniquely combines high accuracy with low and stable runtime, whereas other methods achieve either strong performance with higher computational cost (SynthStrip), lower computational cost with inconsistent accuracy (BET), or a combination of both (ROBEX).

**Fig. 6.**
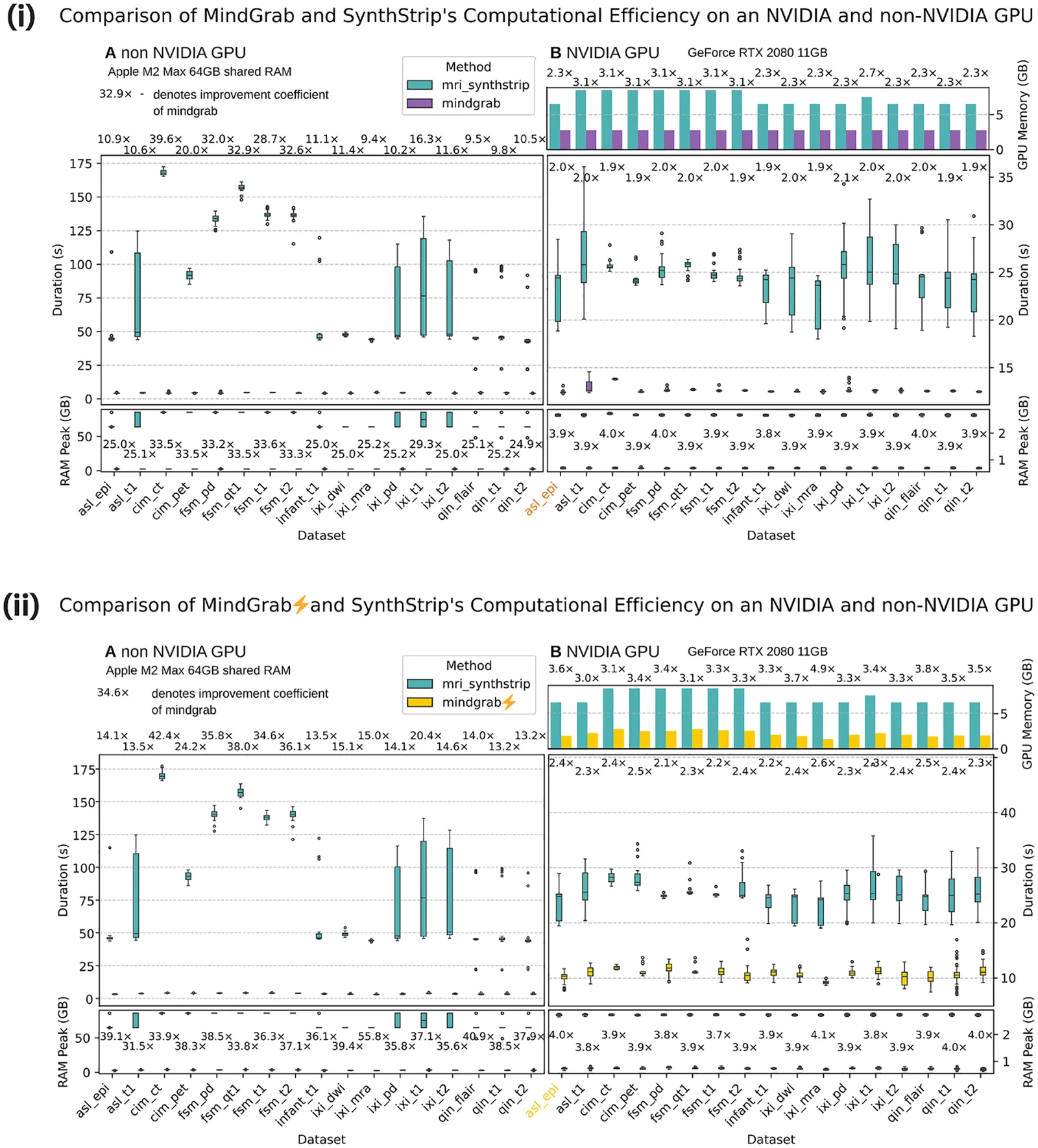
A comparative analysis of MindGrab, MindGrab⚡ and SynthStrip’s computational efficiency for processing the 16 evaluation datasets. Panel (i) compares MindGrab and SynthStrip, and Panel (ii) compares MindGrab⚡ and SynthStrip. Performance is benchmarked for an Apple M2 Max (64GB shared RAM, A) and an NVIDIA GeForce RTX 2080 (11GB, B). The figure compares peak RAM usage (GB, bottom subplot) and run duration (s, middle subplot), with GPU memory usage (GB, top subplot) also shown for the NVIDIA GPU (as GPU memory is shared on Apple M2 Max). Measurements capture the entire pipeline, from loading the NIfTI input to saving the extracted brain output and are based on the command-line implementation of MindGrab and the FreeSurfer version of SynthStrip. Benchmarking used the *pynvml* and *psutil* libraries for the NVIDIA GPU and the *time* utility on the Apple M2 Max to capture memory usage and duration. Multiplicative factors (e.g., 2x) indicate MindGrab[⚡]’s efficiency gain over SynthStrip for each metric.

To provide a more controlled and hardware-aware comparison of computational efficiency, we focus on MindGrab, MindGrab⚡, and SynthStrip, as these models demonstrate competitive segmentation performance and support execution across GPU environments. This enables a more direct evaluation of memory usage and runtime under standardized conditions. We benchmark MindGrab (through brainchopcli), MindGrab⚡ (through brainchop-cli, with the –crop option), and SynthStrip (as mri_synthstrip of FreeSurfer) on an NVIDIA GeForce RTX 2080 GPU 11GB and Apple M2 Max GPU 64GB shared RAM, measuring RAM peak (GB), duration (s), as well as GPU memory (GB) for the NVIDIA GPU (see Fig. 6). We ensure that pre- and post-processing operations guarantee results in the same space for both. On the Apple M2 Max, run duration and RAM usage were captured using the built-in *time* utility. For the NVIDIA GPU, we used the *pynvml* and *psutil* Python libraries to record the relevant metrics. Note that on the Apple M2 Max, GPU memory is shared with the main system RAM; therefore, this metric was not measured separately. Multiplicative factors are used to show efficiency gains and were determined by calculating the ratio of the medians of the two distributions being compared.

Panel (i) results consistently demonstrate MindGrab’s lower RAM peak, shorter execution times, and reduced GPU memory usage compared to SynthStrip. On the NVIDIA GeForce RTX 2080, MindGrab achieves an approximately 4x lower RAM peak, a 2x speedup in run duration, and 2.3–3.1x lower GPU memory usage. The performance disparity is even more pronounced on the Apple M2 Max GPU, where MindGrab speedups range from 9.4–39.6x greater, and its RAM peak falls by approximately 25–33x compared to SynthStrip.

MindGrab⚡’s performance improvements over SynthStrip are even greater (Panel ii). On the NVIDIA GeForce RTX 2080, MindGrab⚡ achieves up to: 4x lower RAM peak, 2.6x speedup, and 4.9x lower GPU memory usage. On the Apple M2 Max GPU, speedups range from 13.2–42.4x greater, and peak RAM falls by 31.5–55.8x compared to SynthStrip.

### 3.4. Effects of architectural changes on multimodal performance

**Fig. 7.**
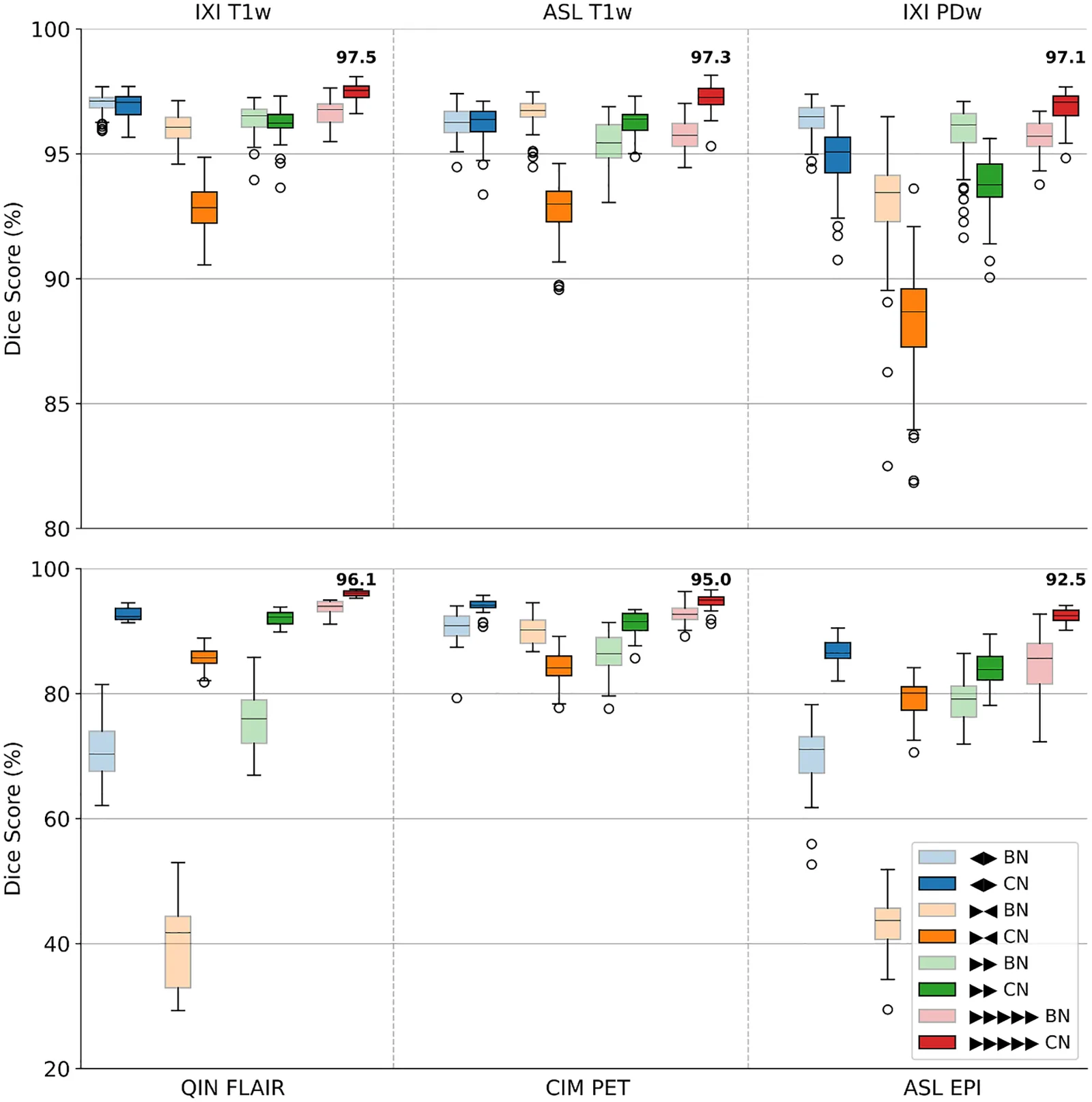
Dice score distributions for architectural configuration and normalization comparison. Dice score distributions for various architectural configurations for six datasets, sampled at the 0th, 20th, 40th, 60th, 80th, and 100th quantiles of MindGrab’s performance (best to worst). Top row (left to right): 0%, 20%, 40%. Bottom row (left to right): 60%, 80%, 100%. Four configurations are shown (◂▸, ▸◂, ▸▸, ▸▸▸▸▸), with ▸ denoting a decreasing dilation sequence (16→8→4→2→1) and ◂ its reverse (1→2→4→8→16). Each configuration includes a BatchNorm (BN) and ChannelNorm (CN) variant. The BN variant is represented by a muted shade of its corresponding CN variant. The median Dice score for MindGrab (▸▸▸▸▸ CN) is displayed above its respective boxplot for each dataset. Note that the y-axis scale differs for the top and bottom figures.

We considered two strategies for combining the blurring and sharpening block sequences defined in Section 2.1: autoencoder-like configurations (◂▸, ▸◂) and stacking identical blocks (▸▸, ▸▸▸▸▸). Fig. 7 displays the performance distribution of these designs with BatchNorm and ChannelNorm variants across six multimodal datasets. ChannelNorm is implemented using Group Normalization (GroupNorm) with the number of groups set equal to the number of channels. This configuration standardizes each channel across the entire spatial extent of each 3D feature map. No learnable affine parameters are applied, making ChannelNorm a parameter-free normalization method. While BatchNorm is commonly used in segmentation literature, our observations indicate that ChannelNorm can lead to substantial performance improvements for MindGrab.

The autoencoder-like configurations exhibited dataset sensitivity. For instance, ◂▸ generally performed well for T1w datasets but struggled with other modalities such as ASL EPI and PDw, while ▸◂ showed greater variability across these datasets. Further refinement of these architectures was challenging due to key practical limitations: autoencoder-like dilation patterns do not lend themselves to effective stacking, and dilations beyond 16 offer no clear advantage (Section 2.1). These insights underscored the utility of the repeated stacking design.

While the ▸▸ model showed competitive performance with ◂▸ for the presented datasets, it did not consistently surpass it. However, the underlying design philosophy of repeated stacking—unreasonable in the autoencoder design—allowed us to extend the architecture further, leading to ▸▸▸▸▸ with parameter-free ChannelNorm, our proposed MindGrab model. MindGrab consistently achieves the highest Dice scores and exhibits the most robust performance among the evaluated configurations.

### 3.5. Evaluation beyond model-derived ground truth

While the results in section 3.1 quantify agreement with the available reference masks, they may not by themselves necessarily establish anatomical correctness. The silver-standard masks provided with the SynthStrip dataset are generated by automated pipelines with subsequent manual corrections, rather than being derived from the aggregation of trusted independent anatomical label maps. Therefore, strong performance relative to these references may reflect alignment with the implicit biases of the models and the human correctors that help produce them (e.g., preference for smoother outer contours versus gyral-following boundaries), rather than fidelity to a meaningful definition of the brain boundary.

To address this limitation, we performed an auxiliary evaluation on the HCP dataset, where high-quality anatomical labels are available. The labels are still silver-standard as they were obtained by FreeSurfer but the data is of an acceptably high quality and we expect fewer inconsistencies. The HCP dataset does not provide a ground truth skull stripping mask. Any mask that covers the brain and does not extend beyond the intracranial volume may be considered acceptable. To address this ambiguity, we construct two reference regions: a brain mask obtained by merging all brain tissue labels, and an intracranial mask defined as the union of brain tissue and surrounding cerebrospinal fluid up to the inner table of the skull. Together, these define an anatomically bounded tolerance region within which acceptable skull stripping outputs should lie–capturing all brain tissue while avoiding inclusion beyond the intracranial boundary.

We evaluate predictions using two criteria against two reference regions. *Recall is computed against the brain mask*: the fraction of true brain voxels included in the prediction. *Precision is computed against the intracranial mask*: the fraction of predicted voxels that fall within the intracranial boundary. Under this formulation, an ideal mask covers all brain tissue (high recall) and stays within the intracranial envelope (high precision).

This experiment reframes the problem from agreement with model-derived masks to compliance with anatomically defined regions, providing a more principled basis for assessing skull stripping quality. We evaluate all four models on 1,112 HCP T1-weighted scans and further assess robustness through a series of controlled perturbations, consisting of resolution degradation, contrast inversion, Rician noise injection, and 90° rotation about the z-axis. For resolution degradation, volumes were downsampled to 3 mm isotropic resolution using trilinear interpolation (nearest-neighbor for labels) and subsequently resampled to 1 mm isotropic resolution to enforce a consistent 256^3^ dimension.

**Fig. 8.**
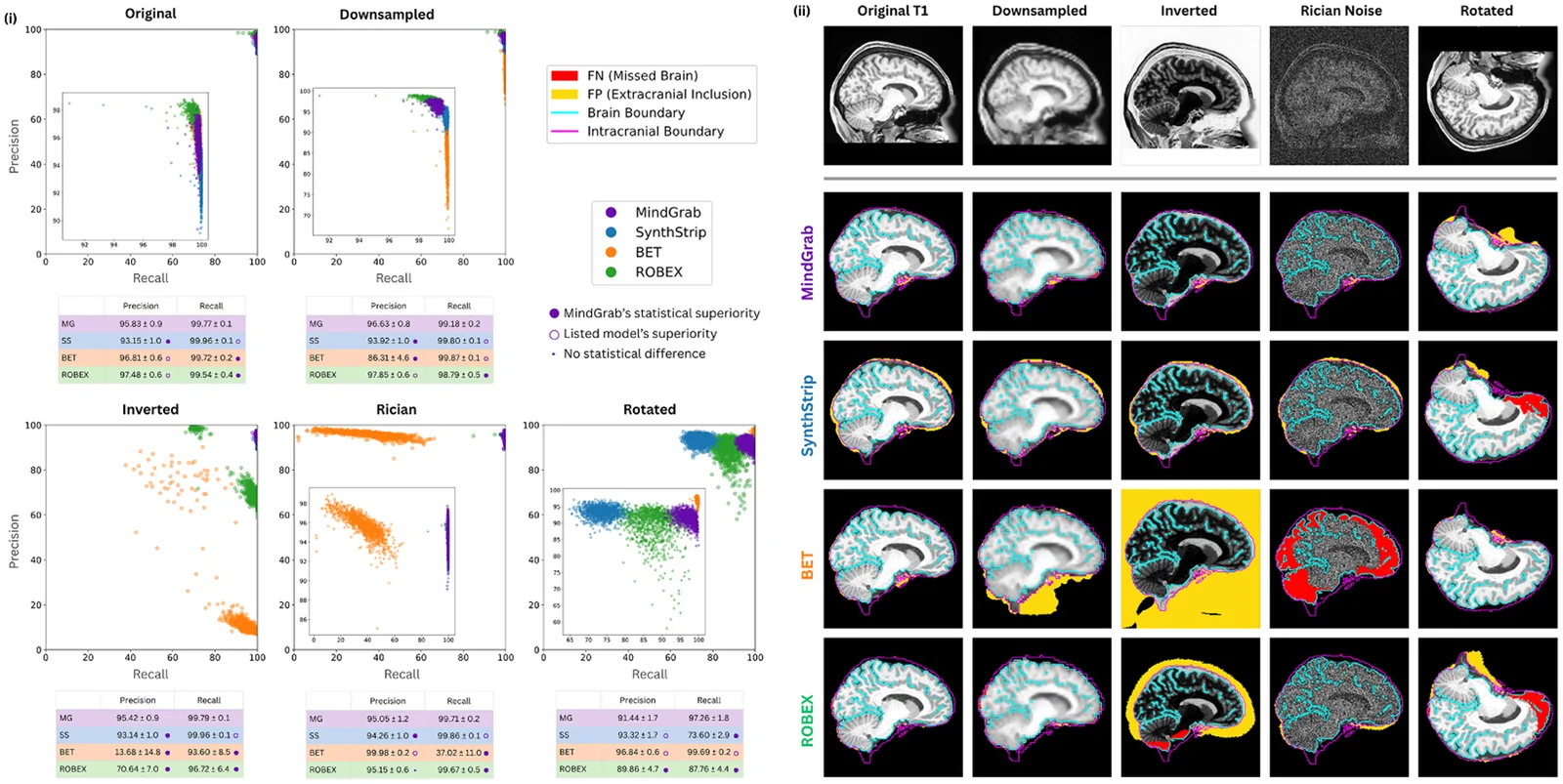
Quantitative and qualitative evaluation of model performance on HCP T1w dataset. Panel (i) presents precision-recall scatterplots for each stress condition (original, downsampled, inverted contrast, Rician noise, and rotated), with precision on the y-axis and recall on the x-axis. All plots share the same scale; inset views are included where necessary to better visualize clustered distributions. Each plot is accompanied by a table reporting mean ± standard deviation of precision and recall for each model, with dots indicating statistical significance of MindGrab relative to other methods based on two-sided Wilcoxon signed-rank tests. MindGrab is represented in purple, SynthStrip blue, BET orange, and ROBEX green. Panel (ii) provides qualitative comparisons. Columns correspond to each perturbation condition, with the first row showing the input images for a representative subject. Subsequent rows display model outputs, with ground-truth brain and intracranial boundaries overlaid in cyan and magenta, respectively. False negatives relative to the brain mask (missed brain tissue) are shown in red, and false positives relative to the intracranial mask (extracranial inclusion) are shown in yellow.

Fig. 8 summarizes these results. Panel (i) presents precision-recall plots for each stress condition, with MindGrab shown in purple, SynthStrip in blue, BET in orange, and ROBEX in green. Accompanying tables report mean ± standard deviation for both metrics, with dots indicating the significance of MindGrab scores as determined by two-sided Wilcoxon signed-rank tests. Panel (ii) provides qualitative visualizations of model behavior under each perturbation. Each column corresponds to one condition, with the first row showing the perturbed input image. Subsequent rows display model outputs, with ground-truth brain and intracranial boundaries overlaid in cyan and magenta, respectively. Two error types are visualized: false negatives with respect to the brain mask indicating missed brain tissue (red), and false positives with respect to the intracranial mask indicating extracranial inclusion (yellow).

In the original condition, all models achieve near-perfect recall with minimal variance in precision. SynthStrip attains the lowest precision (93.15 ± 1.5), while MindGrab, BET, and ROBEX achieve higher values by approximately 2–4 points. Despite these differences, performance across models is comparable visually and tightly clustered near the top-right of the plot. This trend persists under downsampling, with the exception of BET, which exhibits an approximately 10-point drop in precision, indicating increased extracranial inclusion visually evident as large regions of false positives (yellow) in Panel (ii).

The inverted contrast condition produces a pronounced separation. MindGrab and SynthStrip remain tightly clustered at high precision-recall values, whereas BET and ROBEX experience an approximately 80- and 37-point drop in precision, respectively. BET’s high recall in this setting can be misleading as the model tends to capture the majority of the image volume rather than isolating brain tissue, which is evident from the extensive yellow region. ROBEX similarly overcaptures by a wide band surrounding the brain.

Under Rician noise, MindGrab, SynthStrip, and ROBEX show comparable performance with moderate dispersion. BET exhibits a marked reduction in recall, missing large portions of the brain as indicated by false negatives in the qualitative examples.

Finally, in the 90° rotation about the z-axis, each model forms a distinct cluster. BET retains near-original precision and recall. MindGrab maintains high recall (97.26 ± 1.8) with slightly reduced precision (91.44 ± 1.7), indicating mild over-segmentation. In contrast, SynthStrip achieves higher precision but substantially lower recall (93.32 ± 1.7, 73.60 ± 2.9), reflecting under-segmentation. ROBEX shows greater variability in both metrics, with recall falling between MindGrab and SynthStrip. These behaviors are reflected qualitatively, where SynthStrip and ROBEX miss portions of the brain and MindGrab, SynthStrip, and ROBEX overextend.

Overall, these results confirm that MindGrab maintains a balanced high precision-recall score with controlled variability. SynthStrip shows a tendency toward high recall with lower precision, visible as a consistent yellow band surrounding its output masks. Classical methods perform comparably on the original, ‘clean’ data but degrade under distribution shifts, indicating reduced robustness. These anatomically-grounded results corroborate earlier findings on the SynthStrip benchmark, demonstrating that MindGrab’s high performance is not merely agreement with model-derived references.

**Fig. 9.**
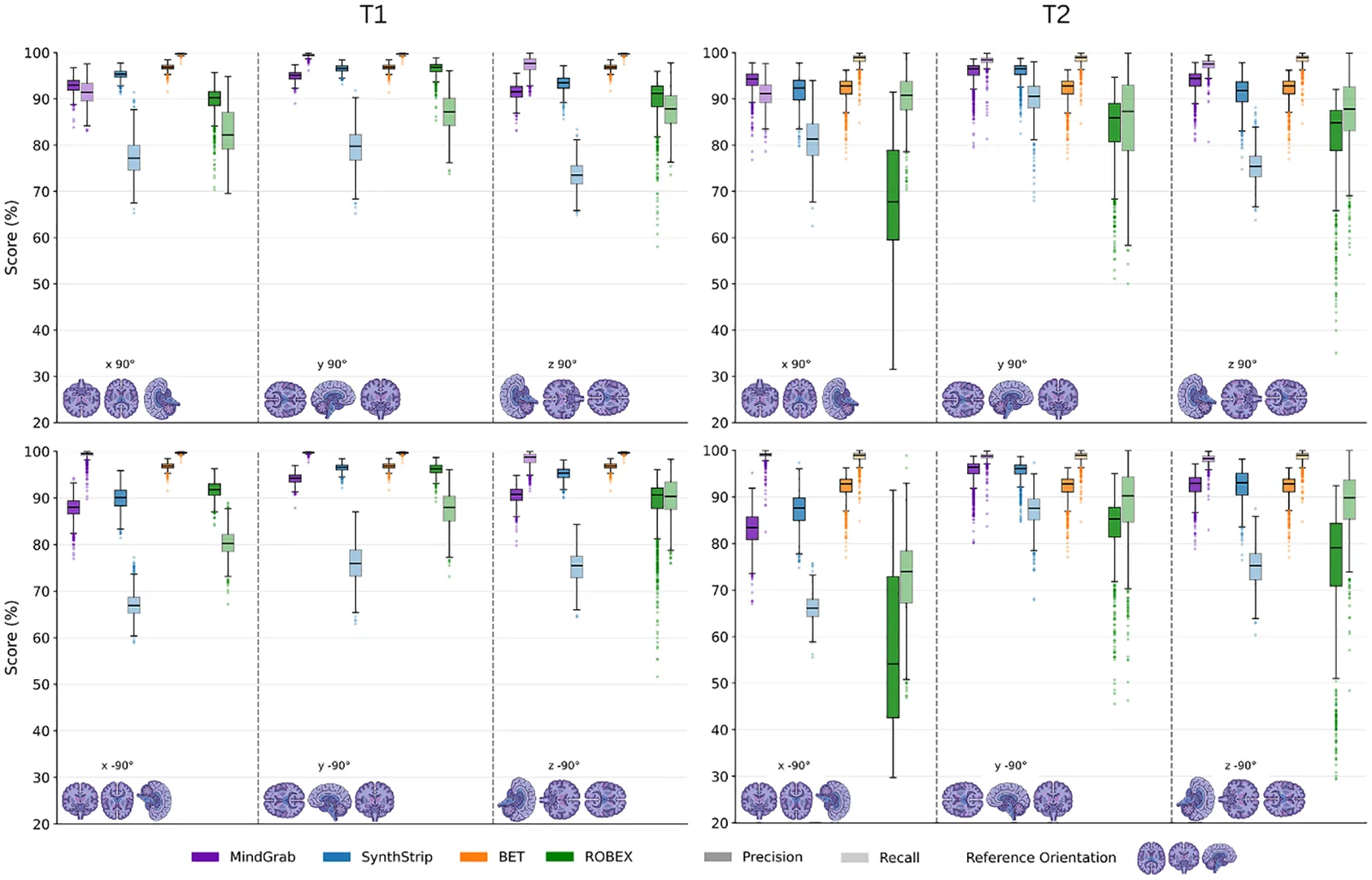
Dice performance across single-step 90° rotations. Boxplots show the distribution of Dice scores for each model under all six single-step 90° rotations (±90° about the x, y, and z axes). T1w results are displayed on the left and T2w on the right. MindGrab is shown in purple, SynthStrip in blue, BET in orange, and ROBEX in green, with recall indicated by a lighter shade of each model’s color. Brain icons at the bottom left of each plot illustrate the resulting orientation after each rotation.

The rotation orientation for the analysis in Fig. 8 was arbitrarily selected. Therefore, we further assess robustness to orientation by evaluating performance across all six single-step 90° rotations (±90° about the x, y, and z axes). Fig. 9 presents boxplot distributions of precision and recall for each model on HCP T1 and T2 data under each rotation. MindGrab boxplots are shown in purple, SynthStrip in Blue, BET in orange, and ROBEX in green, with recall indicated by the lighter shade of each color.

MindGrab maintains high precision and recall across all rotations and both modalities, with a slight decrease in performance and increased variance for rotations about the x-axis. This robustness is notable given that standard CNNs are not inherently rotation-invariant, suggesting that the model has learned features that generalize well across orientation changes. SynthStrip exhibits stable precision but shows reduced recall compared to MindGrab and its own unrotated performance, indicating a tendency to miss portions of the brain.

In contrast, BET exhibits nearly identical performance across all rotations, signifying that its behavior is largely insensitive to orientation. This invariance likely arises from its reliance on intensity-based and geometry priors rather than learned spatial features, resulting in consistent high performance. ROBEX shows the greatest variability, particularly on T2 data, with substantial relative performance degradation and a large number of outliers.

In summary, MindGrab achieves strong robustness to single-step orientation changes, while SynthStrip shows moderate sensitivity, and classical methods either remain invariant with high performance (BET) or exhibit great instability (ROBEX).

## 4. Discussion

Skull stripping remains a common yet challenging preprocessing step in neuroimaging pipelines. Our model, MindGrab, was designed with both accuracy and deployment feasibility as primary objectives. Its constant low memory footprint and low parameter count enable deployment as a lightweight command-line tool and in-browser model. We publicly release both versions through BrainChop under a permissive MIT license. The model (581 KB) and full command-line package (<20 MB with dependencies) are one-click installable on macOS, Linux, and Windows via brainchop-cli. The browser version requires no setup and can be accessed at brainchop.org. The model is identical across both platforms, ensuring that high accuracy is consistent regardless of deployment method. brainchop.org offers an intuitive user interface for single-instance analysis and quality control. The zero-footprint convenience of the browser version involves a performance trade-off, with runtimes that are longer (although still competitively fast) than the command-line tool and dependent on local hardware. The command-line tool is a better fit for large-scale data processing, leveraging its superior efficiency and support for batch operations to deliver significantly higher throughput than SynthStrip.

The performance advantage is particularly pronounced on non-NVIDIA hardware, such as Apple Silicon, demonstrating the tool’s optimization for modern computing platforms beyond the specialized GPU ecosystem. The joint runtime-Dice analysis clarifies the trade-offs between tools, with important emphasis on their efficiency. BET offers extremely fast execution and can perform sufficiently on some datasets, but lacks consistency and degrades under atypical image characteristics. ROBEX demonstrates somewhat greater performance across modalities but still has substantial variability in accuracy and longer runtimes than BET, though it should be noted that ROBEX runtimes were measured on a separate Linux system and are not hardware-normalized with the other tools. SynthStrip achieves consistently high segmentation accuracy with a similarly high computational cost. In contrast, MindGrab provides an ideal combination of the two: high accuracy with low runtime and reduced variability in both. This advantage is critical, as access to specialized GPUs remains limited in many research and clinical settings. MindGrab’s lightweight architecture and high performance on common hardware, therefore, remove a primary bottleneck to the adoption of advanced AI tools. Ultimately, this democratizes access to state-of-the-art brain extraction, enabling its widespread and practical use in biomedical applications.

MindGrab’s evaluation scores across evaluated datasets reveal its performance characteristics. MindGrab exhibits higher average precision than recall, indicating a conservative boundary delineation approach. In practice, this behavior suggests that MindGrab is more likely to minimally undersegment the brain compartment (as seen in the SynthStrip qualitative masks), prioritizing the accuracy of identified brain voxels. In contrast, MindGrab⚡ (cropped input) shifts this balance, leading to higher recall scores with a slight reduction in precision. This change implies a modified segmentation strategy more effective at capturing the whole brain, but with an increased propensity for including some non-brain tissue. This balance is aligned with SynthStrip’s performance, which also generally favors recall over precision (overshoots) across most modalities. The availability of this adjustable trade-off between precision and recall through the default and cropped options of MindGrab’s command-line implementation offers valuable flexibility for users, allowing them to select the mode that best fits their specific application and timing preference.

Qualitatively, MindGrab demonstrates highly effective and robust skull stripping for CT, PET, and MR scans, with its errors being uncommon and minimal across the evaluated samples. The primary difference between the model’s prediction and the ground truth is typically over- and under-segmentation of the brain boundary. While these deviations can be genuine errors, they also frequently highlight the inherent ambiguity and subjectivity in defining a precise brain boundary, a challenge common to all skull stripping methods. To resolve undersegmentation differences and provide users with greater control, the brainchop-cli implementation of MindGrab includes a –border flag that allows users to increase the masks’ boundary threshold in millimeters. This functionality ensures that users can achieve their desired segmentation while still benefiting from MindGrab’s core advantages.

Segmentation in MindGrab does not require prior spatial normalization or registration, as the model operates on input images in the subject space. The only transformation applied before MindGrab is an internal conformation step that resamples volumes to a standardized 256^3^ grid before mapping predictions back to the original space. This is particularly relevant for clinical and intraoperative settings, where images may exhibit irregular orientations or lack standardized alignment. Our rotation experiments demonstrate that MindGrab maintains stable performance under 90° rotations, indicating practical robustness to orientation variability. However, in some cases, reorienting images to a canonical orientation (e.g., RAS) may further improve skull stripping quality, particularly in challenging or atypical acquisitions. While registration can be beneficial for downstream analyses that require spatial correspondence across subjects, these results suggest that it is not a prerequisite for accurate skull stripping with MindGrab, reducing preprocessing complexity.

Our design prioritizes a spectral perspective over spatial receptive field size, which offers little practical guidance for architectures of this scale. The high-to-low dilation sequence (▸: 16→8→4→2→1) is key: its initial high-dilation layers are sensitive to high-frequency boundary details, while the subsequent low-dilation layers force this information into a progressively coarser, more robust representation. Cascading five such blocks (▸▸▸▸▸) creates a powerful information bottleneck, compelling the network to learn a highly efficient representation suitable for memory-constrained deployment. This design is complemented by the use of ChannelNorm (GroupNorm with groups equal to channels), a parameter-free standardization that empirically yielded greater robustness than standard batch normalization.

While MindGrab performed competitively against SynthStrip and outperformed ROBEX and BET on the tested benchmarks, it has limitations with infant populations and segmenting images with artifacts that have high-contrast boundaries. The exclusion of pediatric data from training and validation, combined with challenges of infant skull stripping—including age-specific anatomical differences and lower tissue contrasts—leave pediatric performance unverified and may limit the generalizability of the method to younger populations. Future research will aim to either train a dedicated pediatric version of MindGrab or develop a synthetic data generation protocol that more accurately captures the unique anatomical trends of infant populations. In addition, given the emphasis on accessibility and deployment, future studies will aim to evaluate the acceptability of MindGrab in clinical settings, including integration into existing workflows and user interactions with the tool. Nevertheless, its favorable performance efficiency trade-off makes MindGrab a practical tool for clinical and research applications.

## Supplementary Material

1

Supplementary material associated with this article can be found, in the online version, at doi:10.1016/j.neuroimage.2026.122074.

## Figures and Tables

**Table 1:** Dice comparison of skull stripping models ↑.

Modalities	MindGrab⚡	MindGrab	SynthStrip	ROBEX	BET
FSM T1w	97.6 ± 0.3 ^°^	97.5 ± 0.3	97.8 ± 0.3 ^°^	96.0 ± 0.7 •	66.8 ± 8.9 •
IXI T1w	97.3 ± 0.4 •	97.5 ± 0.4	97.1 ± 0.5 •	96.1 ± 0.8 •	88.2 ± 6.9 •
FSM qT1	97.5 ± 0.4 ^°^	97.4 ± 0.4	97.7 ± 0.2 ^°^	81.7 ± 11.8 •	68.2 ± 4.0 •
ASL T1w	96.8 ± 0.6 •	97.3 ± 0.5	97.3 ± 0.5 ·	96.8 ± 1.1 •	89.4 ± 5.7 •
FSM T2w	97.3 ± 0.5 ^°^	97.1 ± 0.5	97.8 ± 0.3 ^°^	93.0 ± 1.8 •	92.0 ± 5.7 •
IXI T2w	97.1 ± 0.7 ^°^	97.0 ± 0.7	96.6 ± 0.5 •	91.4 ± 2.6 •	92.5 ± 3.5 •
IXI PDw	97.2 ± 0.7 ^°^	96.9 ± 0.6	96.7 ± 0.5 •	94.5 ± 1.3 •	94.7 ± 1.9 •
IXI MRA	94.1 ± 1.3 •	96.7 ± 0.7	97.4 ± 0.5 ^°^	73.9 ± 8.4 •	86.7 ± 9.5 •
FSM PDw	96.9 ± 0.5 ^°^	96.4 ± 0.5	97.6 ± 0.3 ^°^	95.6 ± 1.0 •	87.4 ± 6.9 •
QIN FLAIR	96.1 ± 0.4 ·	96.0 ± 0.5	96.0 ± 0.5 ·	93.0 ± 4.1 •	95.4 ± 1.2 •
CIM CT	95.7 ± 1.5 ·	95.9 ± 1.2	95.3 ± 1.0 •	73.8 ± 2.4 •	41.5 ± 4.4 •
QIN T1w	94.6 ± 2.0 ·	94.9 ± 1.5	95.8 ± 1.0 ^°^	92.9 ± 3.6 •	92.8 ± 3.1 •
CIM PET	95.0 ± 1.3 ^°^	94.7 ± 1.4	95.0 ± 1.0 ·	91.9 ± 3.5 •	89.2 ± 5.9 •
IXI DWI	94.0 ± 1.4 ^°^	93.7 ± 1.7	95.6 ± 0.9 ^°^	87.2 ± 6.3 •	93.9 ± 1.3 ·
QIN T2w	93.4 ± 1.9 ·	93.5 ± 1.8	95.0 ± 1.1 ^°^	87.1 ± 6.7 •	89.3 ± 4.4 •
ASL EPI	92.3 ± 1.1 ·	92.4 ± 0.9	95.2 ± 1.0 ^°^	80.8 ± 6.4 •	94.6 ± 1.1 ^°^

• = MindGrab’s statistical superiority

° = listed model’s superiority

· = no statistical difference.

Table 1**. Skull Stripping Performance Comparison Across Multimodal Datasets**. Metrics show the average Dice score ± standard deviation between computed and ground-truth brain masks. Rows are ordered by descending MindGrab Dice score. MindGrab is applied to 256^3^ inputs. MindGrab and MindGrab⚡ achieve comparably competitive or superior performance, significantly outperforming ROBEX universally and BET in most cases, with mixed results against SynthStrip.

## Data Availability

MindGrab is publicly and freely available for use on the browser (brainchop.org) and command-line (brainchop-cli). Both versions are released under a permissive open source MIT license. Benchmark datasets used for evaluation are publicly accessible and detailed in the manuscript.
