# Supplementary material for: MindGrab: A spectrally-motivated architecture for accessible deep learning in neuroimaging
Source: Neuroimage. Author manuscript; Available in PMC 2026 Aug 31. (PMC13527664; doi:10.1016/j.neuroimage.2026.122074)
Supplement: Supplementary file 1 [file NIHMS2200352-supplement-1.docx]

**Supplement**

**Table S1:** Mean Surface Distance to the Ground Truth $\downarrow$

| **Modality** | **MindGrab** | **MindGrab**⚡︎ | **SynthStrip** | **ROBEX** | **BET** |
| --- | --- | --- | --- | --- | --- |
| FSM T1w | 1.1 ± 0.1 | 1.1 ± 0.1 | 1.0 ± 0.1 | 1.7 ± 0.3 | 16.2 ± 4.8 |
| IXI T1w | 1.1 ± 0.1 | 1.2 ± 0.1 | 1.3 ± 0.2 | 1.5 ± 0.3 | 5.1 ± 3.0 |
| FSM qT1 | 1.1 ± 0.1 | 1.1 ± 0.1 | 1.0 ± 0.1 | 8.0 ± 5.8 | 15.9 ± 2.2 |
| ASL T1w | 1.2 ± 0.2 | 1.4 ± 0.2 | 1.2 ± 0.2 | 1.3 ± 0.4 | 4.3 ± 2.4 |
| FSM T2w | 1.3 ± 0.2 | 1.2 ± 0.2 | 1.0 ± 0.1 | 2.9 ± 0.9 | 3.5 ± 2.6 |
| IXI T2w | 1.2 ± 0.3 | 1.2 ± 0.3 | 1.4 ± 0.2 | 3.5 ± 1.2 | 2.9 ± 1.3 |
| IXI PDw | 1.3 ± 0.2 | 1.2 ± 0.3 | 1.4 ± 0.2 | 2.1 ± 0.5 | 2.2 ± 0.8 |
| IXI MRA | 1.2 ± 0.2 | 2.3 ± 0.5 | 1.0 ± 0.1 | 11.0 ± 4.0 | 4.5 ± 3.1 |
| FSM PDw | 1.5 ± 0.2 | 1.3 ± 0.2 | 1.1 ± 0.1 | 1.8 ± 0.4 | 5.8 ± 3.2 |
| QIN FLAIR | 1.5 ± 0.2 | 1.5 ± 0.1 | 1.5 ± 0.2 | 2.7 ± 1.8 | 1.7 ± 0.4 |
| CIM CT | 1.8 ± 1.0 | 2.0 ± 1.3 | 1.8 ± 0.4 | 11.5 ± 1.4 | 38.6 ± 4.7 |
| QIN T1w | 2.0 ± 0.6 | 2.1 ± 0.8 | 1.6 ± 0.4 | 2.7 ± 1.5 | 2.7 ± 1.2 |
| CIM PET | 2.0 ± 0.6 | 1.9 ± 0.5 | 1.9 ± 0.4 | 3.5 ± 1.5 | 4.5 ± 2.8 |
| IXI DWI | 2.4 ± 0.6 | 2.3 ± 0.6 | 1.7 ± 0.4 | 5.1 ± 2.8 | 2.3 ± 0.5 |
| QIN T2w | 2.4 ± 0.7 | 2.4 ± 0.7 | 1.8 ± 0.4 | 5.1 ± 3.1 | 3.9 ± 1.7 |
| ASL EPI | 2.6 ± 0.4 | 2.7 ± 0.4 | 1.6 ± 0.3 | 7.8 ± 3.1 | 1.9 ± 0.4 |

**Table S1**: Symmetric Mean Surface Distance (MSD ± standard deviation) between predicted and ground-truth brain masks for MindGrab, MindGrab⚡︎ (cropped input), SynthStrip, ROBEX, and BET across 16 diverse datasets. MindGrab, MindGrab⚡︎, and SynthStrip achieve low MSD values, indicating similarly accurate boundary delineation. ROBEX and BET show greater variability across modalities.

Table S1 reports the Symmetric Mean Surface Distance (MSD) to ground truth masks for each dataset, complementing the Dice scores and precision/recall relationships presented in Table 1 and Figure 1 of the main text. MSD is computed as the average of the minimum distance from each surface voxel in one mask to the surface voxels in the other mask, evaluated in both directions. While previously discussed metrics assess the volumetric overlap between predicted and ground truth masks, MSD quantifies how close the predicted brain boundary is to the ground truth boundary, highlighting boundary irregularities that might be averaged out in volumetric scores.

MindGrab, MindGrab⚡︎, and SynthStrip consistently achieve low MSD scores, indicating high boundary accuracy and surface conformity. SynthStrip has slightly lower values across several of the more challenging datasets, such as ASL EPI and IXI DWI, but both MindGrab and MindGrab⚡︎ remain within approximately one voxel of its performance. In contrast, ROBEX and BET scores exhibit substantial variability across datasets. While they perform competitively in isolated cases (e.g. BET on IXI DWI and ASL EPI; ROBEX on ASL T1w), their overall inconsistency highlights limitations in generalizing across imaging contrasts and modalities.

**Table S2:** 95^th^ Percentile Symmetric Hausdorff Distance (HD95) $\downarrow$

| **Modality** | **MindGrab** | **MindGrab**⚡︎ | **SynthStrip** | **ROBEX** | **BET** |
| --- | --- | --- | --- | --- | --- |
| FSM T1w | 2.9 ± 0.4 | 2.7 ± 0.4 | 2.9 ± 0.4 | 4.6 ± 0.8 | 71.9 ± 16.1 |
| IXI T1w | 3.0 ± 0.7 | 3.0 ± 0.6 | 3.7 ± 0.6 | 4.7 ± 0.9 | 29.8 ± 17.3 |
| FSM qT1 | 3.0 ± 0.3 | 2.9 ± 0.3 | 2.9 ± 0.3 | 19.0 ± 10.9 | 75.7 ± 11.1 |
| ASL T1w | 3.0 ± 0.7 | 3.2 ± 0.7 | 3.4 ± 0.8 | 3.9 ± 2.2 | 21.8 ± 12.6 |
| FSM T2w | 3.2 ± 0.4 | 3.0 ± 0.4 | 2.8 ± 0.4 | 7.2 ± 3.5 | 17.5 ± 13.5 |
| IXI T2w | 3.2 ± 0.9 | 3.1 ± 0.8 | 3.8 ± 0.7 | 8.7 ± 3.1 | 9.2 ± 4.1 |
| IXI PDw | 3.3 ± 0.7 | 3.1 ± 0.8 | 3.7 ± 0.6 | 5.6 ± 1.9 | 8.2 ± 5.3 |
| IXI MRA | 3.3 ± 0.8 | 7.7 ± 2.4 | 2.6 ± 0.6 | 22.6 ± 4.4 | 13.8 ± 7.2 |
| FSM PDw | 3.6 ± 0.4 | 3.3 ± 0.4 | 3.0 ± 0.3 | 4.6 ± 1.6 | 35.8 ± 15.9 |
| QIN FLAIR | 4.3 ± 0.6 | 4.1 ± 0.5 | 4.4 ± 0.5 | 6.7 ± 4.2 | 5.1 ± 1.6 |
| CIM CT | 6.2 ± 7.0 | 8.1 ± 11.2 | 5.7 ± 1.5 | 20.5 ± 5.4 | 137.2 ± 15.5 |
| QIN T1w | 6.1 ± 2.4 | 7.1 ± 4.4 | 4.9 ± 1.5 | 8.3 ± 3.6 | 9.7 ± 4.9 |
| CIM PET | 5.1 ± 2.0 | 5.0 ± 1.9 | 5.3 ± 1.6 | 7.9 ± 2.4 | 15.0 ± 10.6 |
| IXI DWI | 6.7 ± 2.4 | 6.9 ± 2.3 | 4.8 ± 1.2 | 13.4 ± 5.9 | 7.1 ± 1.9 |
| QIN T2w | 8.3 ± 2.9 | 9.1± 3.6 | 5.7 ± 1.8 | 12.9 ± 5.5 | 15.2 ± 6.9 |
| ASL EPI | 7.7 ± 1.1 | 8.0 ± 1.3 | 4.6 ± 1.0 | 18.4 ± 4.0 | 4.9 ± 1.0 |

**Table S2**: 95th Percentile Symmetric Hausdorff Distance (HD95) across 16 imaging datasets spanning different modalities for the five evaluated brain extraction methods. MindGrab, MindGrab⚡︎, and SynthStrip achieve low HD95 scores, whereas ROBEX and BET achieve highly variable and large HD95 scores.

We report the 95^th^ Percentile Symmetric Hausdorff Distance (HD95) in Table S2 to evaluate worst-case boundary errors and each model’s robustness to localized segmentation failures. HD95 provides an upper bound on the geometric error encountered in 95% of all boundary points, quantifying the severity of the largest segmentation error while mitigating the influence of isolated outliers.

MindGrab, MindGrab⚡︎, and SynthStrip consistently achieve lower HD95 scores. Performance between MindGrab and MindGrab⚡︎ is generally comparable, with each showing slight advantages on different datasets consistent with trends observed in other evaluation metrics. Conversely, ROBEX and BET exhibit substantial variability and large HD95 scores across most modalities and datasets, reflecting inconsistent performance and reduced generalizability.
